# Thermal stress reduces dung burial, brood production, and the development of dung beetles: a challenge for climate resilience

**DOI:** 10.1093/ee/nvag104

**Published:** 2026-11-05

**Authors:** Zinat Jahan Chowdhury, Mohammed Rashed Chowdhury, Zac Hemmings, Karl Vernes, Timothy M Schaerf, Nigel R Andrew, Alfonsina Arriaga-Jiménez

**Affiliations:** School of Environmental and Rural Science, University of New England, Armidale, NSW, Australia; Department of Entomology, Sylhet Agricultural University, Sylhet, Bangladesh; School of Environmental and Rural Science, University of New England, Armidale, NSW, Australia; Department of Biochemistry and Chemistry, Sylhet Agricultural University, Sylhet, Bangladesh; School of Environmental and Rural Science, University of New England, Armidale, NSW, Australia; Department of Primary Industries, Coffs Harbour, NSW, Australia; Department of Regional NSW, Coffs Harbour, NSW, Australia; School of Environmental and Rural Science, University of New England, Armidale, NSW, Australia; School of Science and Technology, University of New England, Armidale, NSW, Australia; School of Environmental and Rural Science, University of New England, Armidale, NSW, Australia; Entomology Lab, Faculty of Science and Engineering, Southern Cross University, Lismore, NSW, Australia; School of Science and Technology, University of New England, Armidale, NSW, Australia; Queen Victoria Museum and Art Gallery, Launceston, Tasmania, Australia

**Keywords:** climate change, competition, dung utilization, heat stress, reproduction

## Abstract

Increasing temperatures can impair ecosystem services and the reproduction of dung beetles. We investigated the effects of increased nest temperatures on reproduction, dung burial, and the development of 2 dung beetle species (*Onthophagus binodis* and *Digitonthophagus gazella*). Three pairs of sexually mature F1-beetles at 15 days of age were housed in temperature-controlled chambers for 2 weeks. Temperature treatments of +3 °C and +5 °C above baseline (control: a diurnal temperature cycle modelled from 2019 to 2023 summer data at the beetles origin location) were applied. Fresh cattle dung was provided to feed the beetles, and the dung burial was recorded weekly. The number of broods produced, brood success, developmental days, pronotum width, and body weight of F2 progeny were recorded. Relative to the baseline treatment, brood production was reduced by 43.1% at +3 °C and 39.3% at +5 °C, with no significant difference between the 2 elevated-temperature treatments. Dung burial was 96 g/kg dry matter lower when beetles were reared at +3 °C compared to the control. Increased temperature did not affect brood success; however, the highest nest temperature reduced the development time of *D. gazella* by 2.45 days compared to the control or the +3 °C treatment. In addition, a mixed effect of the highest temperature was observed in the pronotum width, which was 0.49 mm smaller and 0.32 mm greater in the F2 beetles of *O. binodis* and *D. gazella*, respectively, compared to those that were reared with +3 °C. In conclusion, heat stress negatively affected brood construction, reduced dung burial and development of F2 progeny, and altered pronotum width, suggesting that climate warming may compromise dung beetle-mediated ecosystem services such as dung removal and associated nutrient cycling.

## Graphical Abstract

**Graphical Abstract:**
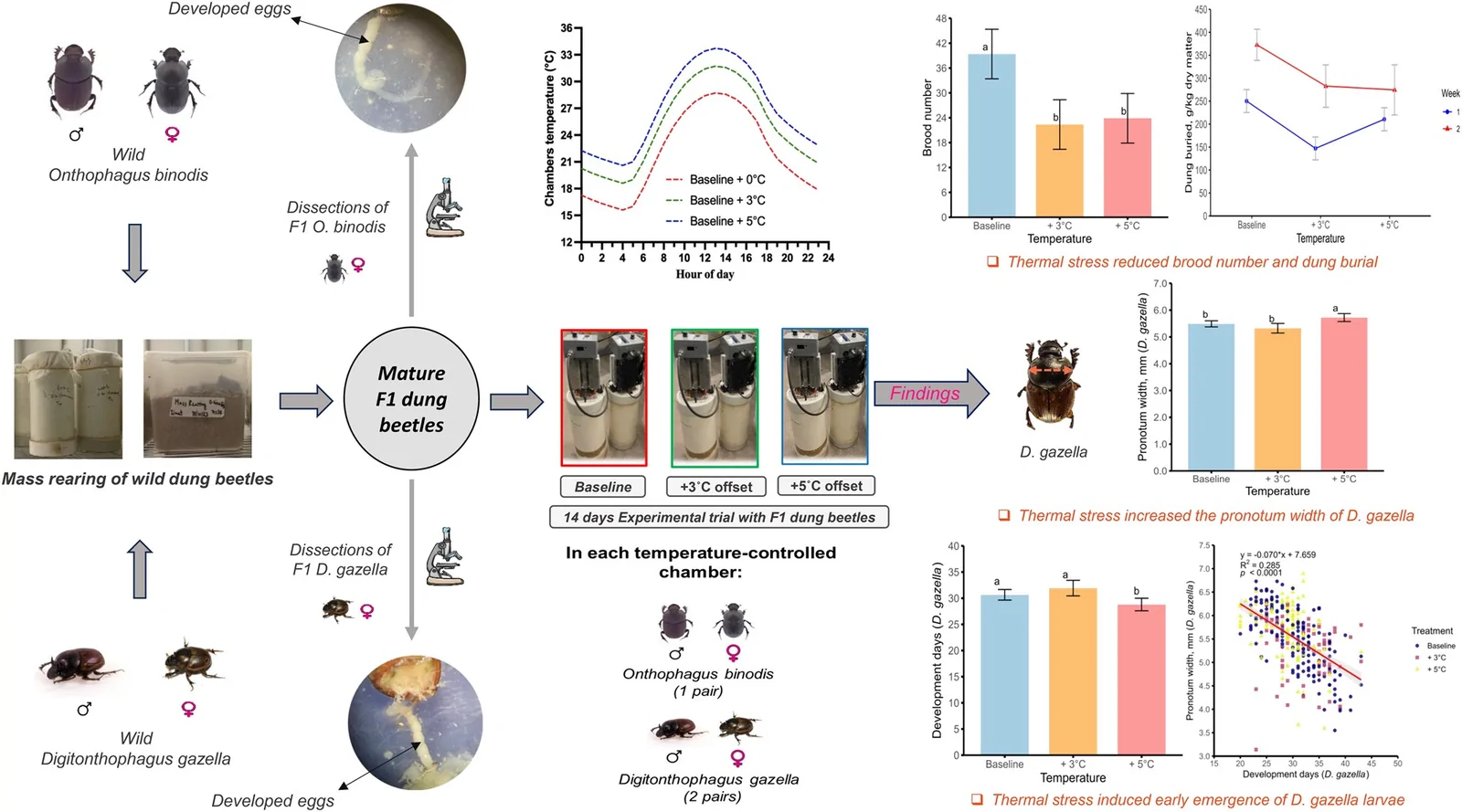

## Introduction

As the world’s climatic conditions change rapidly, it becomes more important to understand how species adapt and react to these changes, which significantly affects insect behavior and ecology, including species extinction, shifts in species assemblages, compositions, interactions with other species, and species abundance and distribution (Pecl et al. 2017, Schleuning et al. 2020, Bhagarathi and Maharaj 2023). Among the consequences of climate change, rising temperatures are expected to impose substantial physiological and ecological challenges on insects because their performance is closely linked to environmental temperature (Urban et al. 2012). However, insect species vary considerably in their thermal tolerance and responses to warming, which may influence their survival, reproduction, and ecological functions, ultimately altering the structure and functioning of terrestrial ecosystems (Andrew and Hill 2017, Ma et al. 2021).

Dung beetles (Coleoptera: Scarabaeidae) are active ecosystem engineers because their dung removal and nesting activities modify soil properties and contribute to several ecosystem services, including soil aeration, secondary seed dispersal, dung decomposition, pollination, nutrient cycling, pest control, and greenhouse gas reduction (Nichols et al. 2008, Doube 2018, deCastro-Arrazola et al. 2023, Holley et al. 2026). Adult beetles feed on dung, whereas larval development depends on the species’ nesting strategy. For instance, in paracoprid species, adults excavate tunnels beneath the dung pat and provision underground brood chambers with dung for larval development, whereas endocoprid species develop within the dung pat itself (Bogoni and Hernandez 2014). Through dung relocation and burial, paracoprids (tunnelers) and telecoprids (rollers, which transport dung balls away from the original pat before burial) play complementary roles in the dispersal of seeds (Lima et al. 2024) and improve soil hydrological properties by increasing soil porosity and water infiltration (Brown et al. 2010).

Previous studies have shown that thermal stress can affect dung beetle abundance, distribution, and population dynamics (Andrew 2011, Machekano et al. 2021, Gotcha et al. 2022). A warming climate has also been shown to impact dung beetle life history, body size (Wu and Sun 2012), habitat selection (Menéndez and Gutiérrez 2004, Giménez Gómez et al. 2022), species range (Birkett et al. 2018), selection of activity time (Gotcha et al. 2021), dung burial, and reproduction (Holley and Andrew 2019a). According to Holley and Andrew (2020), the dung burial of *Onthophagus taurus* was affected by prolonged warming, which ultimately reduced brood production and population size. Similarly, a warming climate reduced offspring survival in *Onthophagus hecate* (Mamantov and Sheldon 2021). Thermal conditions may also alter reproductive behavior of dung beetles. For instance, female *Phanaeus vindex* produced more, but smaller, broods that were buried deeply under temperatures averaging approximately 2 °C above control conditions (Kirkpatrick and Sheldon 2022). However, Gotcha et al. (2021) reported that the impact of thermal stress on dung beetle activity may depend on the species’ critical thermal maximum (CT_max_) values. The CT_m__a__x_ of dung ­beetles may vary with species. For instance, the CT_m__a__x_ of *Digitonthophagus gazella* (*D. gazella*) was slightly higher than that of *Onthophagus binodis* (*O. binodis*), at 48.5 °C (Gotcha et al. 2021) compared to 46.43 °C (Hemmings et al. 2019), respectively. Additionally, adult *O. taurus* were 3 times smaller and pupal metabolism was lowest when individuals were reared in a warm, variable treatment (26 ± 8 °C) compared to 22 °C and 24 °C (± 2 °C, ± 4 °C) treatments (Morgan Fleming et al. 2021).

Temperature, resource availability, and biotic interactions are important drivers of dung beetle performance and the ecosystem services they provide (Dell et al. 2014, McConnell and Rohner 2025, McConnell et al. 2026). Previous studies have demonstrated that warming affects dung beetle reproduction, ecosystem services, survivability, and behavioral plasticity using wild-caught individuals (Holley and Andrew 2019a, 2020, Mamantov and Sheldon 2021, Gotcha et al. 2022) or multiple species (Holley and Andrew 2019b). However, variation in the age and body condition of field-collected beetles may confound temperature effects on reproduction and brood development (Hanski and Cambefort 1991, Hunt and Simmons 2001, Bowler and Terblanche 2008, Cotter et al. 2011). In our previous studies using age-controlled first-generation (F1) individuals of *O. binodis* and *D. gazella*, we demonstrated that density and the absolute numbers of female and male beetles in small groups influenced reproductive performance and brood construction (Chowdhury et al. 2026). However, the effects of elevated temperature on reproduction, dung burial, brood success, and second-generation (F2) development, when these 2 species coexist, have not been examined.

Here, we assessed the effects of increasing chamber temperature on brood production, dung burial, brood success, and F2 developmental traits in *O. binodis* and *D. gazella* maintained together under controlled laboratory conditions. We anticipated that increasing habitat temperature would reduce reproductive performance, dung burial, brood success, and F2 development for both species.

## Materials and Methods

The current study was conducted using laboratory-reared F1 individuals of *O. binodis* and *D. gazella* to examine the effects of thermal stress on dung burial, reproduction, and F2 development under controlled laboratory conditions. Three pairs of sexually mature F1 beetles (1 pair of *O. binodis* and 2 pairs of *D. gazella*) were housed together in temperature-controlled chambers for 2 weeks in the Insect Ecology Laboratory, NSW, Australia. The choice of these pairing treatments for both species was based on our previous observations, which showed that a single pair of *O. binodis* and 2 pairs of *D. gazella* produced the highest brood numbers and achieved comparatively greater reproductive success among the group compositions tested in previous work (Chowdhury et al. 2024). The laboratory temperature was maintained at approximately 25 °C, and the relative humidity was maintained at 40%.

### Collection of Cattle Dung, Storage, and Nutrient Composition

Fresh cattle dung was collected early in the morning from the pastureland of Farrer Memorial Agricultural High School, Tamworth, NSW, Australia. Dung was voided by cattle that were fed green grass in the pastureland. During each visit, approximately 60 kg of cattle dung was collected, transferred to the laboratory, homogenized using a mixer machine, and stored in a freezer at −20 °C in 3 l plastic bags. The subsamples of dung from each 3 l bag, primarily used in the current experiment, were pooled, and the pH of the dung at room temperature was recorded using an electric pH meter (SKU: EC-PH6/02K, South Carolina, USA). Another subsample of pooled dung (∼500 g) was dried using a hot-air oven at 55 °C for 5 days until achieving a constant weight by following the AOAC guidelines (AOAC International 2012). A cyclone grinder machine (Retsch-Allee 1–5, Haan, Germany) fitted with a 1.0 mm sieve was used to grind the dried dung, and the subsample was transferred to a commercial laboratory (Feed Central Laboratory, Charlton, QLD 4350, Australia) for nutrient and mineral analysis (Table 1).

**Table 1.** Chemical and mineral composition (expressed as g/kg dry matter unless specified otherwise) of cattle dung fed to dung beetles under controlled laboratory conditions

Item	Dung
**Chemical composition**	
**Moisture (g/kg)**	856
**pH**	7.16
**Dry matter (g/kg)**	144
**Organic matter**	825
**Crude protein**	173
**Neutral detergent fibre**	464
**Acid detergent fibre**	297
**Hemicellulose**	167
**Ash**	175
**Total nitrogen (N)**	27.7
**Total carbon (C)**	413
**C: N**	14.9
**Mineral composition**	
**Potassium**	3.10
**Calcium**	36.4
**Magnesium**	13.1
**Phosphorus**	8.60
**Sodium**	0.60
**Copper (ppm)**	44.0
**Iron (ppm)**	1380
**Manganese (ppm)**	313
**Molybdenum (ppm)**	1.50
**Zinc (ppm)**	130

### Beetles’ Collection and Mass Rearing

Wild beetles (*O. binodis* and *D. gazella*) were collected using dung-baited traps from the same pastureland (Latitude 31˚08ˊ22ˊˊS, Longitude 150˚58ˊ53ˊˊE, Tamworth, NSW, Australia) from which the dung was collected as indicated above. Both species were selected due to their widespread coexistence in NSW, Australia, as well as their effective ecosystem services, such as the removal of large amounts of dung into the soil, which leads to improved nutrient cycling, soil aeration, and seed dispersal compared to dweller and roller dung beetles. The introduced Australian population is referred to here as *D. gazella*, and no further taxonomic assessment was undertaken to distinguish members of the *D. gazella* species complex recognized by Genier and Moretto (2017). Collected beetles were then cleaned using tap water, subsequently separated by sex and species using a light microscope (Leica M60), and prepared for mass rearing.

To align with our research objectives, mass rearing of each field-collected beetle species was conducted in a 9 l plastic container for reproduction. The container was filled with a mixture of moistened vermiculite and sand (2:1), and approximately 350 g of cattle dung was used as feed twice weekly. Ten pairs (10 males + 10 females) of each beetle species were placed immediately on the top of the dung, and the residual dung was removed in subsequent feedings. Fourteen days later, broods were collected and transferred to another 5 l vermiculite container, which was sprayed using a water gun as required, and placed in the temperature-controlled laboratory for 3 to 4 weeks until emergence of the F1 generation. The F1 beetles were then reared for an additional 15 days in a separate 3 l container using the same mass-rearing procedures as field-collected beetles to attain reproductive maturity. Randomly selected F1 females of both species that had been reared with males for 15 days were dissected to confirm reproductive maturity based on the presence of mature eggs in the ovaries (see graphical abstract). Finally, long-horned major males and mature female beetles of both species were randomly selected for the current study due to their higher reproductive performance than the minor males and immature females, to evaluate the thermal effects on dung burial, reproduction and physiology of F2 dung beetles.

### Calculation of Baseline Temperatures

Daily temperature data from 2019 to 2023 were retrieved for the point of origin of our beetle colonies using data recorded by the Australian Bureau of Meteorology at the Tamworth Airport weather station (http://www.bom.gov.au/places/nsw/tamworth; ID: 55325), NSW, Australia. The Australian NSW summer temperature data for the 3 subsequent months (December to February) from each year was used to determine the mean temperature for each hour over a 24-hour period. This hourly temperature regime over a 24-hour period was used as a baseline temperature in the experiment (Fig. 1).

**Fig. 1.**
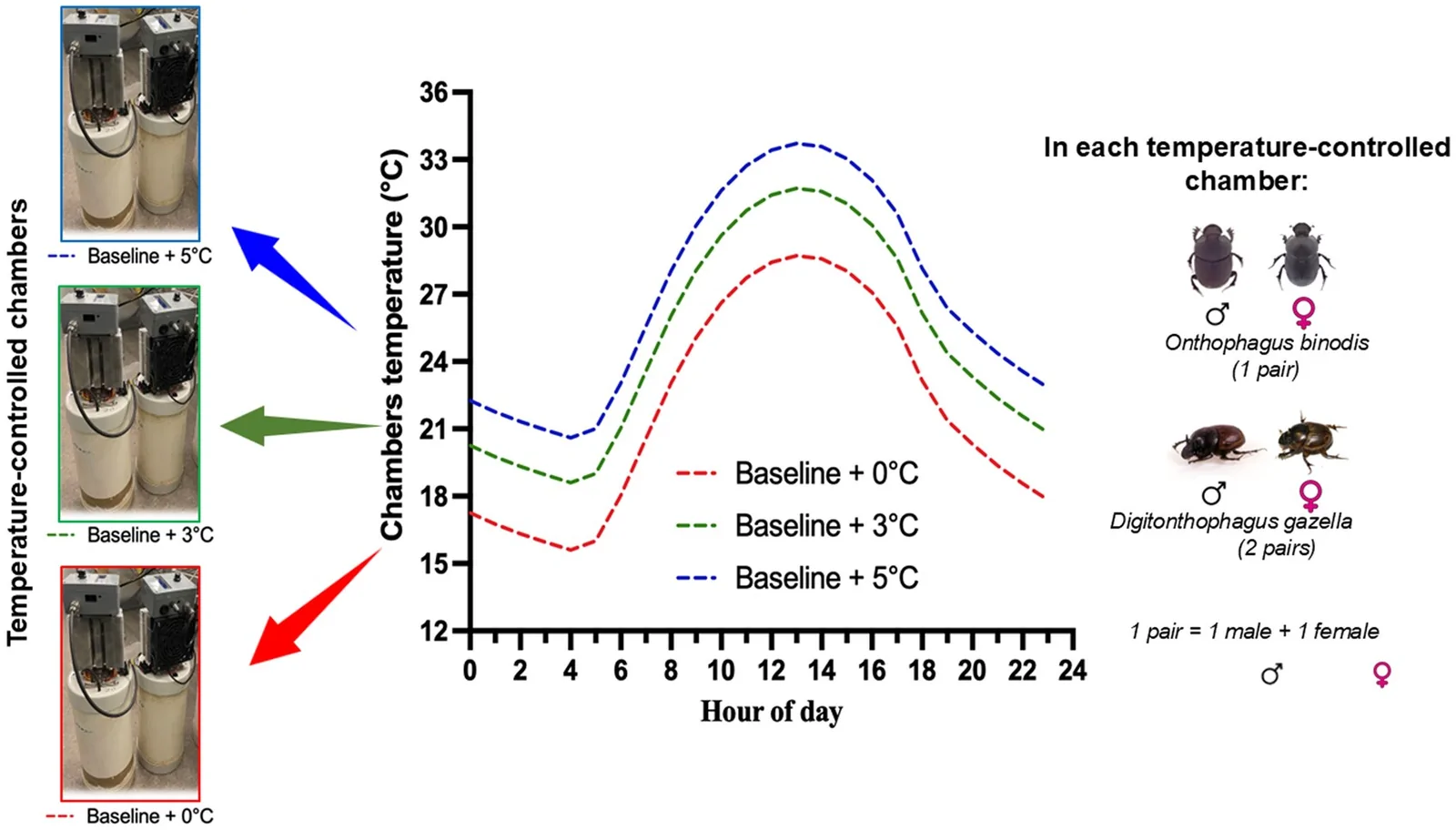
Schematic illustration of the experimental design. Temperature-controlled chambers (both front and back sides) are shown on the left, and the number of beetle pairs (1 pair of *Onthophagus binodis* + 2 pairs of *Digitonthophagus gazella*) in each chamber is displayed on the right. To evaluate the main effect of temperature on dung beetles’ reproduction and performance, we increased the temperature of controlled chambers throughout the experimental period (2 weeks) to add either a + 0 °C (——), +3 °C (——) or +5 °C (——) offset to the calculated 24-hour baseline temperature, as shown in the middle graph. A + 0 °C offset to the baseline temperature was used as a control. Three temperature-controlled chambers were randomly assigned as blocks for 3 offsets, and a total of 24 temperature-controlled chambers (8 blocks × 3 treatments) were used in the experiment.

### Temperature-controlled Chambers

The temperature-controlled chambers used in the experiment were pre-existing infrastructure in the Insect Ecology Laboratory, which had been used in previous dung beetle experiments (Holley and Andrew 2019a, 2019b, 2020). A total of 30 temperature-controlled chambers, including 6 spare chambers, were used in the current experiment to control temperature regimes. Each chamber was built with a PVC plastic tub (height 45 cm, diameter 15 cm), and both open sides were covered with circular PVC lids (Fig. 1). The individual chamber upper lid was fitted with a control, heating, and cooling unit. Each target chamber’s air temperature was controlled by the heating or cooling unit and routinely monitored by a running computer with customized software. The software allowed for setting and tracking each chamber’s air temperature profile (0 to 23 hour period) throughout the experiment. In addition, the chamber’s air temperature was logged by the software to confirm that each chamber’s inside air temperature was tracked every 2 minutes. The chamber’s upper lid was also fitted with lights to provide daylight to the beetles (14 hours; 06:00 to 20:00), which was also controlled by software. Spare lids were used in the experimental trial when the chamber’s cooling or heating unit failed to maintain the set temperature.

### Experimental Design and Treatments

The effect of temperature on dung beetles’ reproduction and performance was assessed by increasing the temperature of controlled chambers throughout the experimental period to add either a + 0 °C, +3 °C, or +5 °C offset to the calculated average 24-hour baseline temperature (Fig. 1). A + 0 °C offset to the baseline temperature was used as a control. Three temperature-controlled chambers were randomly assigned as a block for 3 offsets, and a total of 8 blocks were used as replicates. Therefore, the experiment was conducted using 24 temperature-controlled chambers (8 blocks × 3 treatments). Each chamber (∼ 38 cm height) was filled with a mixture of moistened vermiculite and sand using a similar ratio reported in the mass rearing section. The experimental cattle dung was thawed overnight, and 125 g of wet dung was placed twice weekly on the top of a mixture in each chamber. Additionally, 150 g of wet dung was subsampled during each feeding and stored in the laboratory at −20 °C. Finally, the previously selected F1 beetles, including 1 pair (i.e. pair = 1 male + 1 female) of *O. binodis* and 2 pairs of *D. gazella*, were released together on top of the dung pat of each offset chamber to evaluate the thermal-induced stress on the competition between beetle species for dung burial, brood production, and the development and physiology of F2 progeny. Beetles were then reared in the experimental offset chambers for 2 weeks, allowing beetles to feed and breed. At each feeding time, the residual dung pat was collected, inspected for the presence of any surviving beetles, weighed, and stored in the laboratory for later analyses of organic matter (OM) content.

### Survivability of Beetles, Brood Production, Success Rate, and F2 Progeny

Following 2 weeks of the experimental trial, each chamber with a mixture of vermiculite and sand was carefully inspected for the survivability of parental F1 beetles, and subsequently, the number of broods produced per chamber was recorded without assigning them to species because broods of *O. binodis* and *D. gazella* could not be reliably distinguished morphologically. Broods from each chamber were immediately transferred to a 5 l (32 cm length × 22 cm width × 10 cm height) vermiculite container, placed in the temperature-controlled laboratory for 6 weeks to produce F2 beetles. The vermiculite-containing brood containers were moistened using a water spray bottle and were inspected daily from week 3 onward to receive F2 beetles. The required number of days for F2 beetles to emerge from the brood container was recorded as developmental days or emergence time. Newly emerged day-old F2 adult beetles body weight and pronotum width were measured using a digital balance and electronic slide calipers, respectively. Afterward, the emerged beetles were inspected under a light microscope, and their species identity was recorded for each recovered brood (either *O. binodis* or *D. gazella*) based on the morphological characteristics. Species-specific brood success was then calculated as the number of broods producing F2 individuals of the focal species divided by the total number of broods produced in the corresponding experimental container. However, broods that had not produced F2 adults were examined to determine the cause of brood failure. Unsuccessful broods were identified by the presence of dead larvae rather than unutilized dung or dung balls and were recorded as unsuccessful broods. Finally, the success rate of brood or proportion of F2 progeny, F2 beetles’ developmental days, body weight, and pronotum width were recorded for each species.

### Dung Burial

The collected subsamples of wet dung and the residual dung from the chamber’s dung pat were dried in a hot-air oven at 60 °C to achieve a constant weight by following the AOAC guidelines (AOAC International 2012). The dried dung samples were then milled at a 1.0 mm size using a rotor mill (Retsch-Allee 1–5, Haan, Germany), pooled by feeding week, and analyzed for their OM content. Dung burial of 48 residual samples (2 feeding weeks × 3 treatments × 8 blocks) and 32 subsamples (2 subsamples × 2 feeding weeks × 8 blocks) were calculated using the loss-on-ignition method by following the formula as reported by Bensharada et al. (2022) and Heiri et al. (2001):


$$OM\left(g/kg,dry\;matter\right)=1000-\left(\frac{ash\;weight}{dry\;sample\;weight}\times 1000\right)$$

*Dung buried (g/kg dry matter) = Wet dung OM – Residual dung OM*

The ash weight of dried dung samples was analyzed by following the modified AOAC guidelines using a muffle furnace with the temperature set at 650 °C for 8 hours (AOAC International 2012).

### Statistical Analyses

Statistical analysis was performed via R-Studio version 4.4.3 (R Core Team 2022). Generalized linear mixed-effect models (GLMMs) were used to assess the effects of increasing temperature on brood production, brood success rate, developmental days, pronotum width, and body weight of F2 progeny of *O. binodis* and *D. gazella* via the glmmTMB function from the “*glmmTMB*” package. In addition, the simulateResiduals and testResiduals functions from the “DHARMa” package were applied for residual diagnostics, including assessments for underdispersion and overdispersion (Hartig and Hartig 2017). The analysis of deviance table was created using the Type III Wald chi-square tests. Post hoc analysis was performed with a Holm-Bonferroni adjustment via the emmeans function from the “*emmeans*” package (Lenth et al. 2019). Treatment means were generated using the emmeans function and Holm-Bonferroni adjustment. The data were visualized as boxplots and line graphs using the *ggplot2* package in R. Boxplots show the distribution of the data, with individual observations overlaid where applicable. Significant differences (*P *< 0.05) were denoted by distinct letters within the plots.

The GLMMs were fitted with a Gamma distribution. A log link with a “*Gaussian*” family was applied for models analyzing the developmental days, pronotum width, and body weight of F2 progeny of each species, whereas a “*beta-binomial*” family was used in the models to assess the brood success rate or proportion of F2 progeny. Brood production (count data) was analyzed using a GLMM with a “*Poisson*” distribution and log link function, including treatment as a fixed effect and block as a random effect. For comparisons across treatment means within each species, the block was treated as a random effect, and temperature as a fixed effect.

Dung burial data were normally distributed and were analyzed by a mixed model fitted with a “*lmer*” function from the “*lme4*” package to assess the effect of temperature for both feeding weeks:


$$Y_{\mathrm{ijk}}=\mu +T_{i}+F_{j}+{(T}_{i}{\times F}_{j})+B_{k}+\epsilon_{\mathrm{ijk}},$$

where $Y_{\mathrm{ijk}}$ is the response variable; μ is the overall mean; $T_{i}$ is the temperature effect; $F_{j}$ represents the effect of the feeding period (week 1 and/or 2); $T_{i}{\times F}_{j}$ is the interaction between temperature and feeding period; $B_{k}$ is the effect of block, respectively; and $\epsilon_{\mathrm{ijk}}$ represents the residual error. The effects of temperature, feeding periods, and their interactions were considered fixed effects in the fitted model, while the block was treated as a random effect. Linear regression models were fitted using the *lm* function in R to examine the relationships between development days and pronotum width of F2 progeny, and between brood number and dung burial during feeding weeks 1 and 2. Thermal treatment and its interaction with the continuous predictor were included to test whether the regression relationships differed among treatments. Treatment-specific regressions were also fitted to obtain the regression equations and R^2^ values for graphical presentation. We used similar brood numbers produced by beetles in each chamber for each feeding week while fitting the regression model in R-Studio. Model assumptions were assessed using DHARMa residual and outlier diagnostics, and statistical significance was set at *P *< 0.05. All statistical models, including model fit, residual plots and model summary outputs, are provided in the supplementary material (see supplementary data available online in figshare: https://doi.org/10.6084/m9.figshare.31325461.v3).

## Results

### Survivability of Adult F1 Beetles

All parental F1 beetles in each temperature-controlled chamber, including *O. binodis* and *D. gazella*, remained alive after 2 weeks of the experimental trial. We collected all F1 beetles from the chambers and released them onto ruminant pastureland near the Insect Ecology Laboratory in NSW, Australia.

### Brood Production

We collected a total of 685 broods from the temperature-controlled chambers, which were produced by 2 pairs of *D. gazella* and 1 pair of *O. binodis* dung beetles. Increasing the temperature of a controlled chamber significantly affected (χ^2^ = 37.795, df = 2, *P *< 0.001) the number of broods produced by F1 dung beetles (Fig. 2). Compared to the baseline treatment, increasing the temperature by +3 °C and +5 °C resulted in a 43.1% and 39.3% reduction in brood production, respectively, with no significant difference between the 2 elevated-temperature treatments (mean = 23.2 broods; *P *= 0.630). Both *O. binodis* and *D. gazella* were housed together within the same experimental treatments, and their broods were morphologically similar. Therefore, individual broods could not be reliably assigned to species before F2 emergence. Species-specific outcomes were then investigated after F2 emergence by recording the developmental days of F2 progeny, the number of emerging F2 beetles and the brood success rate for each species.

**Fig. 2.**
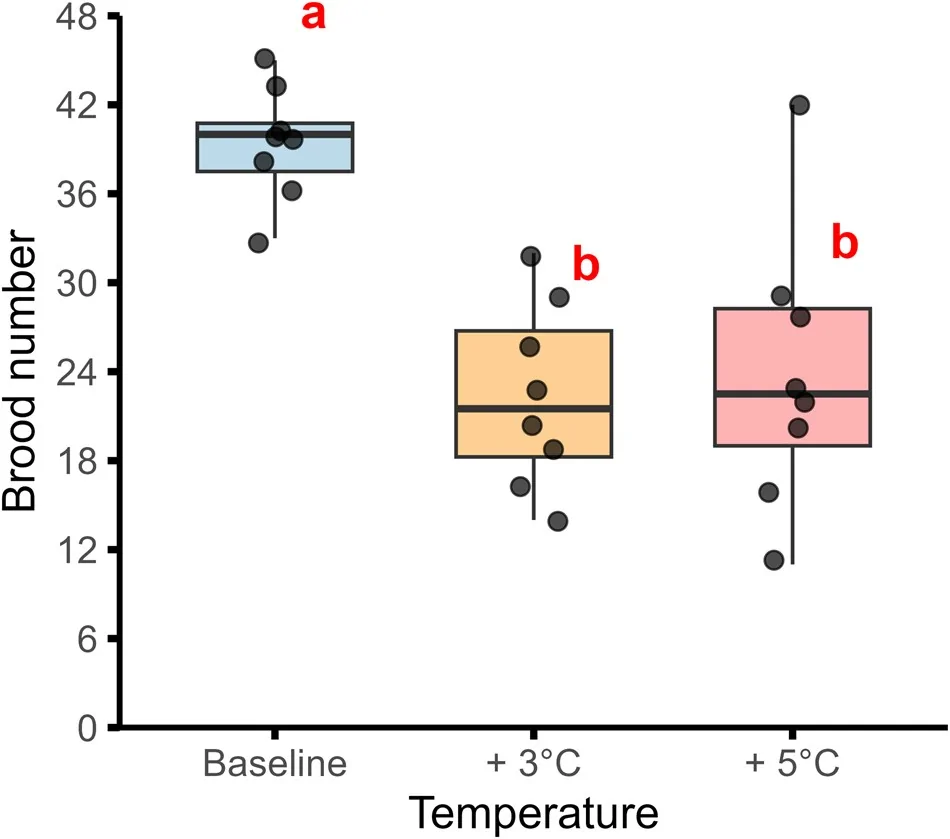
Effect of temperature on brood production by F1 dung beetles. Boxplots show the distribution of brood number, with individual points representing observations. Different letters within the boxplot indicate statistically significant differences (*P* < 0.05). Effect of temperature, χ^2^ = 37.795, df = 2, and *P* < 0.001.

### Developmental Days of F2 Progeny

The required number of days for the emergence of F2 beetles was recorded for each species. This was on average 37.6 ± 4.85 and 30.3 ± 4.91 across the treatments for *O. binodis* and *D. gazella*, respectively. We did not find an effect of temperature (χ^2^ = 2.288, df = 2, *P *= 0.319) on F2 developmental days for *O. binodis* (Fig. 3A). However, temperature affected (χ^2^ = 19.379, df = 2, *P *< 0.001) the required number of days for the emergence of *D. gazella* (Fig. 3B). Increasing the temperature up to +5 °C reduced the F2 developmental days of *D. gazella*, where F2 beetles emerged comparatively 2.45 days earlier than those beetles that were reared at baseline or in a + 3 °C chamber. There was no significant difference in F2 developmental days between *D. gazella* reared in baseline conditions and those reared in a + 3 °C chamber (*P *= 0.071), with an average developmental time of 31.25 days for these treatments.

**Fig. 3.**
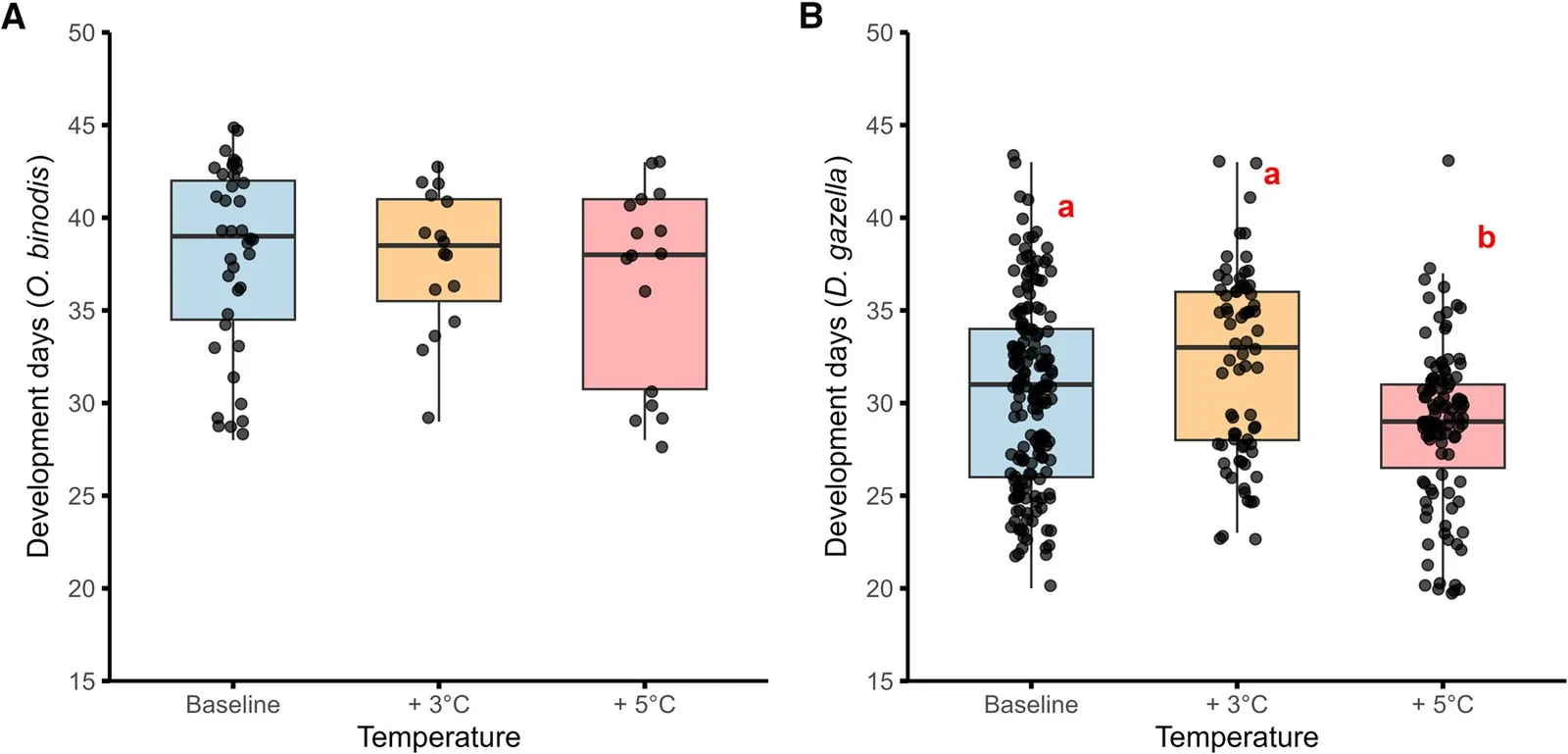
Effect of temperature on development days of *Onthophagus binodis* (A) and *Digitonthophagus gazella* (B). Boxplots show the distribution of individual observations, and points represent individual F2 beetles. Different letters within the boxplot indicate statistically significant differences (*P* < 0.05). For *O. binodis*, χ^2^ = 2.288, df = 2, and *P *= 0.319; and for *D. gazella*, χ^2^ = 19.379, df = 2, and *P* < 0.001.

### Brood Success Rate or Proportion of F2 Progeny

We found 428 newborn F2 beetles across the treatments (baseline = 217, +3 °C offset = 88, and +5 °C offset = 123) and species (*O. binodis *= 72 and *D. gazella *= 356), with an average brood success rate of 62.5%. Unsuccessful broods were attributable to larval mortality, as dead larvae were observed during examination after F2 emergence. The proportion of F2 progeny is shown in Fig. 4, including the proportion of F2 *O. binodis* and *D. gazella*. Temperature treatment did not affect the proportion of F2 *O. binodis* (χ^2^ = 2.076, df = 2, *P *= 0.354; Fig. 4A) or *D. gazella* (χ^2^ = 3.100, df = 2, *P *= 0.212; Fig. 4B), with their average proportion rate being 0.094 and 0.508, respectively. Regardless of species, the numerically highest number of F2 progeny emerged from the baseline chamber, with an average proportion of 0.350.

**Fig. 4.**
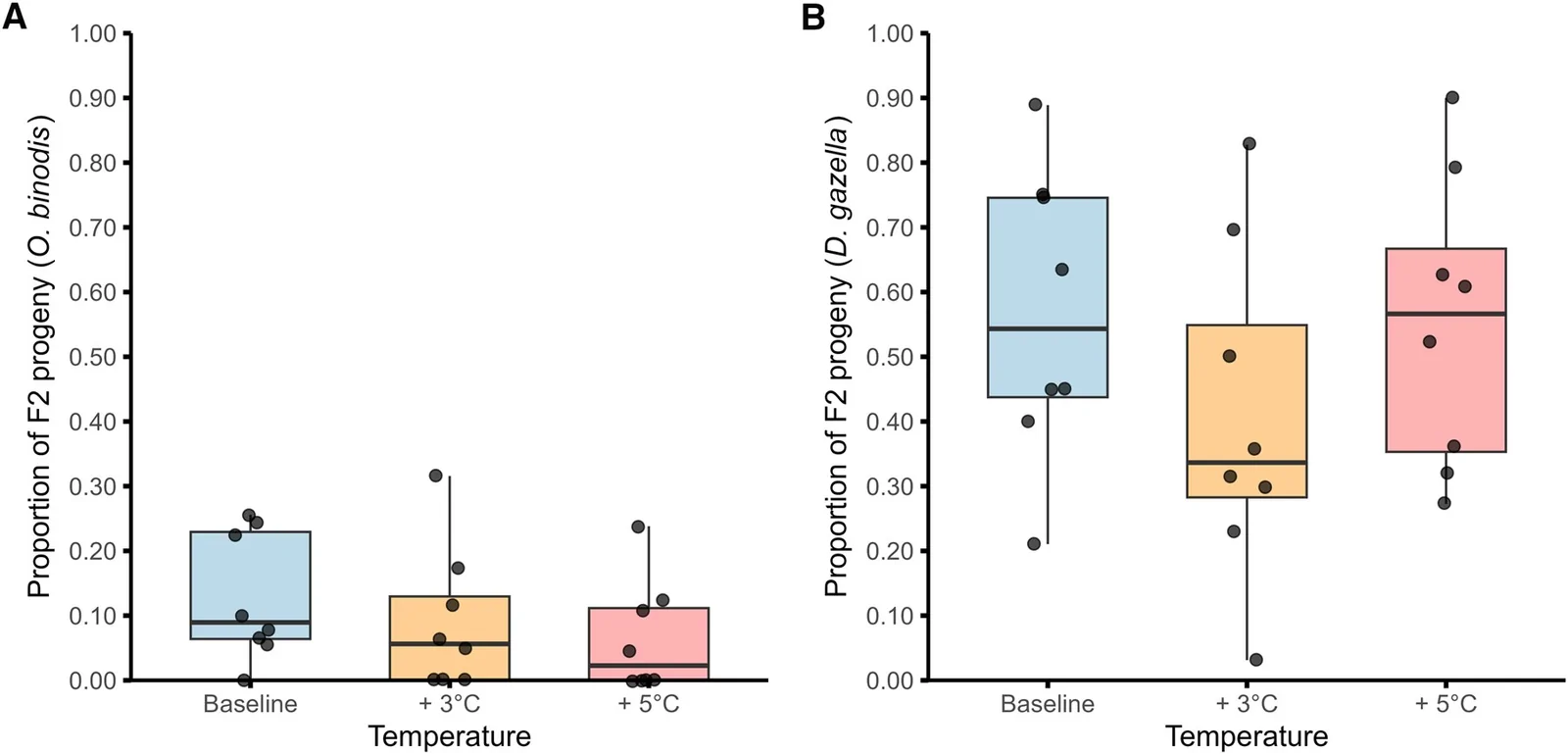
Effect of temperature on brood success rate (proportion of F2 progeny) of *Onthophagus binodis* (A) and *Digitonthophagus gazella* (B). Boxplots show the distribution of observed proportions, with individual points representing observations. For *O. binodis*, χ^2^ = 2.076, df = 2, and *P *= 0.354; and for *D. gazella*, χ^2^ = 3.100, df = 2, and *P *= 0.212.

### Pronotum Width and Body Weight of F2 Progeny

Temperature treatment affected the width of the pronotum in F2 progeny of both species (Fig. 5). For instance, the pronotum width was 0.49 mm smaller (χ^2^ = 6.210, df = 2, *P *= 0.045) in F2 *O. binodis* when F1 beetles were reared in the +5 °C chamber compared to those reared in the +3 °C chamber; however, those beetles reared at baseline temperature had an intermediate value, with an average pronotum width of 5.53 mm (Fig. 5A). In contrast, the pronotum width of F2 *D. gazella* was 0.32 mm greater (χ^2^ = 18.421, df = 2, *P *< 0.001) when the temperature was set at +5 °C compared to those beetles reared in the baseline or +3 °C chamber, which did not vary (*P *= 0.063) and had an average pronotum width of 5.41 mm (Fig. 5B). In addition, development time was negatively associated with pronotum width, but the strength of this relationship varied among temperature treatments (Fig. 6). In *O. binodis*, the relationship was significant only under +5 °C (*R^2^* = 0.273, *P *= 0.038), whereas in *D. gazella* significant negative relationships were observed under baseline (*R^2^* = 0.515, *P *< 0.001) and +5 °C (*R^2^* = 0.137, *P *< 0.001) treatments.

**Fig. 5.**
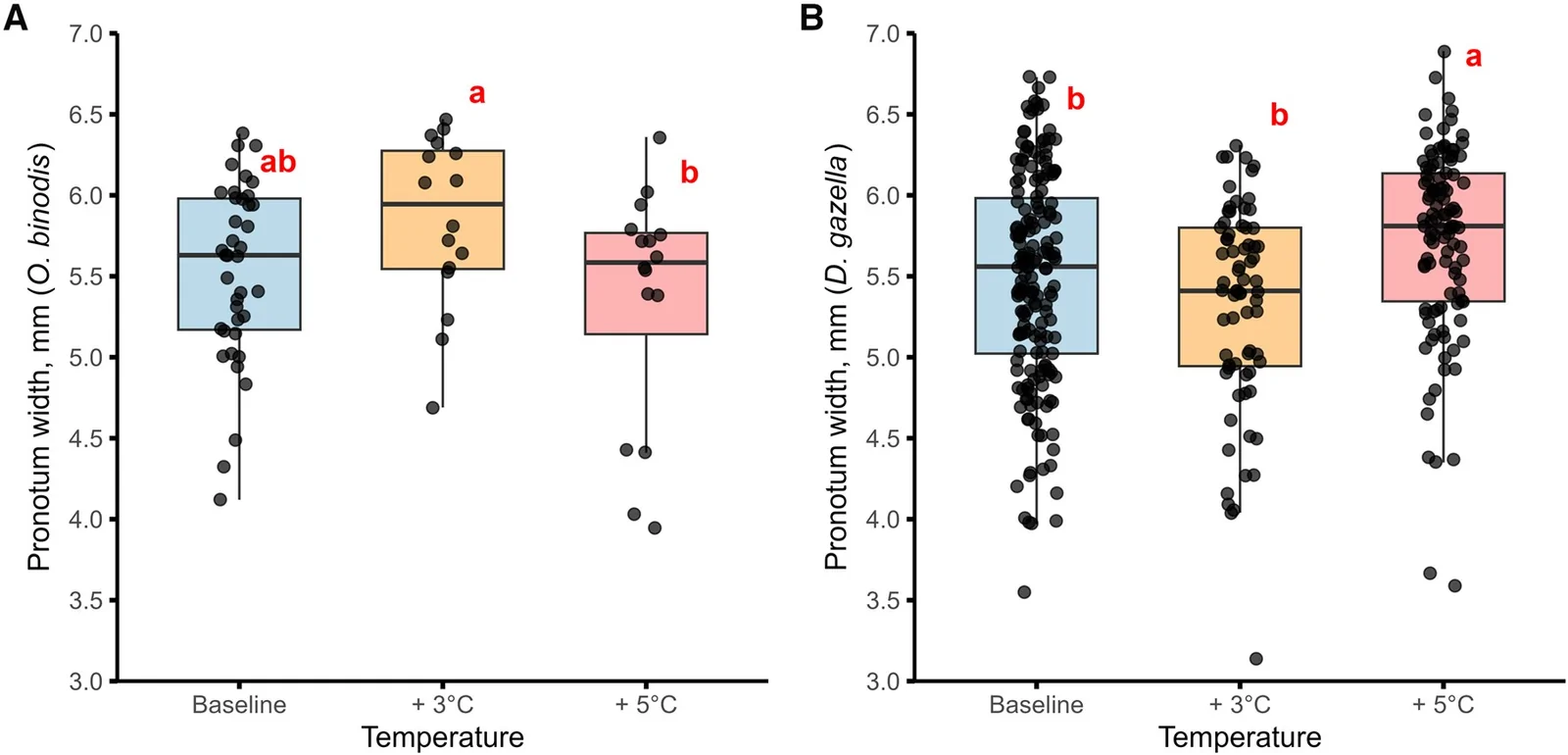
Effect of temperature on pronotum width (mm) of F2 progeny of *Onthophagus binodis* (A) and *Digitonthophagus gazella* (B). Boxplots show the distribution of pronotum width, and points represent individual F2 beetles. Different letters within each boxplot indicate statistically significant differences (*P* < 0.05). For *O. binodis*, χ^2^ = 6.210, df = 2, and *P *= 0.045; and for *D. gazella*, χ^2^ = 18.421, df = 2, and *P* < 0.001.

**Fig. 6.**
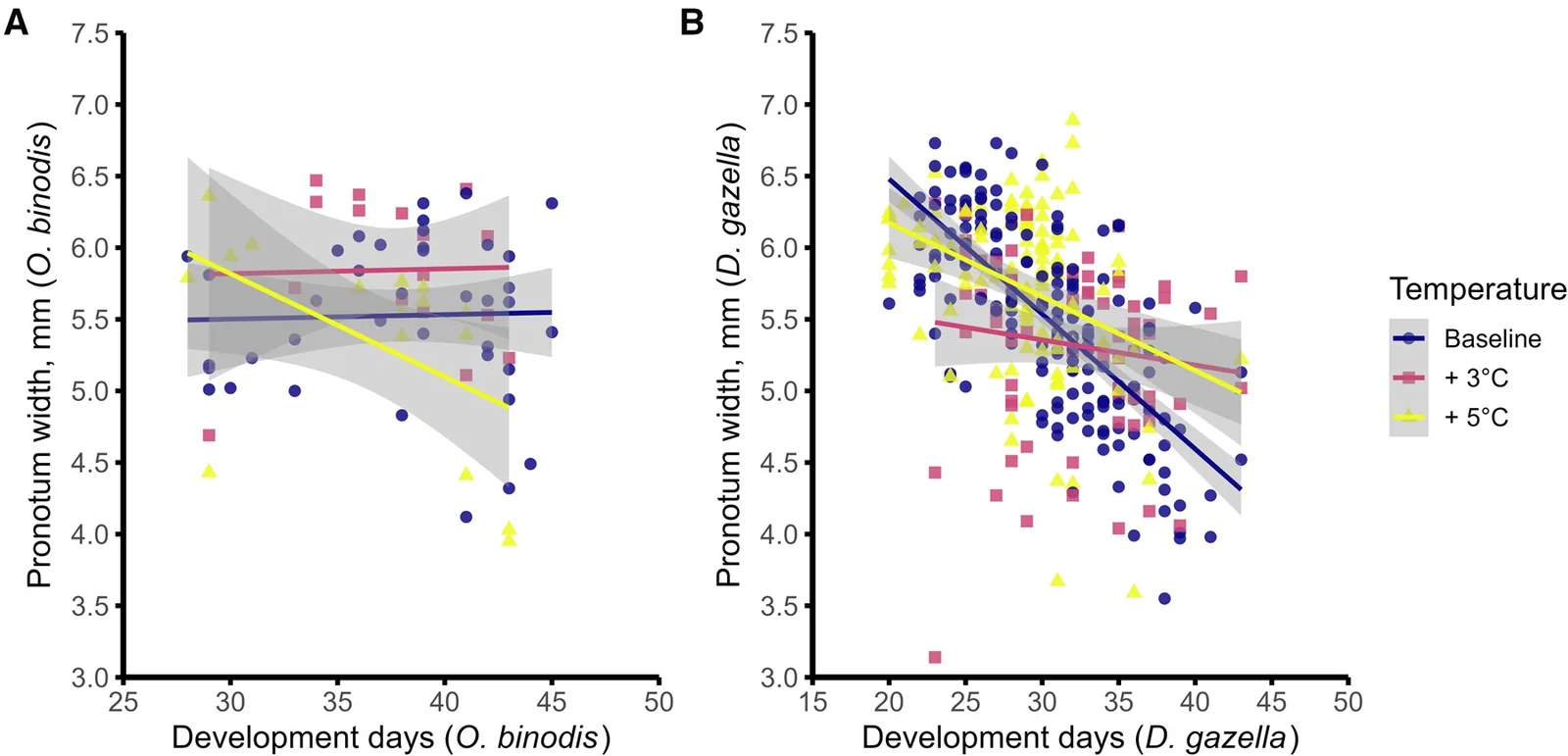
Relationship between the development days and the pronotum width of F2 progeny of *Onthophagus binodis* (A) and *Digitonthophagus gazella* (B) under 3 different temperature treatments. Points represent individual observations, and colored lines represent treatment-specific linear regressions with 95% confidence intervals (shaded areas). For *O. binodis* (A), the regression equations were y = 5.409 + 0.003x (baseline; R^2^ = 0.001, *P *= 0.862), y = 5.718 + 0.003x (+3 °C; R^2^ = 0.001, *P *= 0.928), and y = 7.980 − 0.072x (+5 °C; R^2^ = 0.273, *P *= 0.038). For *D. gazella* (B), the regression equations were y = 8.365 − 0.094x (baseline; R^2^ = 0.515, *P* < 0.001), y = 5.883 − 0.018x (+3 °C; R^2^ = 0.019, *P *= 0.247), and y = 7.203 − 0.052x (+5 °C; R^2^ = 0.137, *P* < 0.001).

Increasing the baseline temperature by +3 °C or +5 °C did not affect (χ^2^ = 1.539, df = 2, *P *= 0.463) the body weight of F2 progeny of *O. binodis*, which was on average 89.0 mg (Fig. 7A). Similarly, the body weight of F2 *D. gazella* was not affected (χ^2^ = 3.929, df = 2, *P *= 0.140) by the temperature treatment, which averaged 69.0 mg (Fig. 7B). However, regardless of the treatments, the body weight of F2 *O. binodis* was numerically 20 mg heavier than that of the F2 *D. gazella*.

**Fig. 7.**
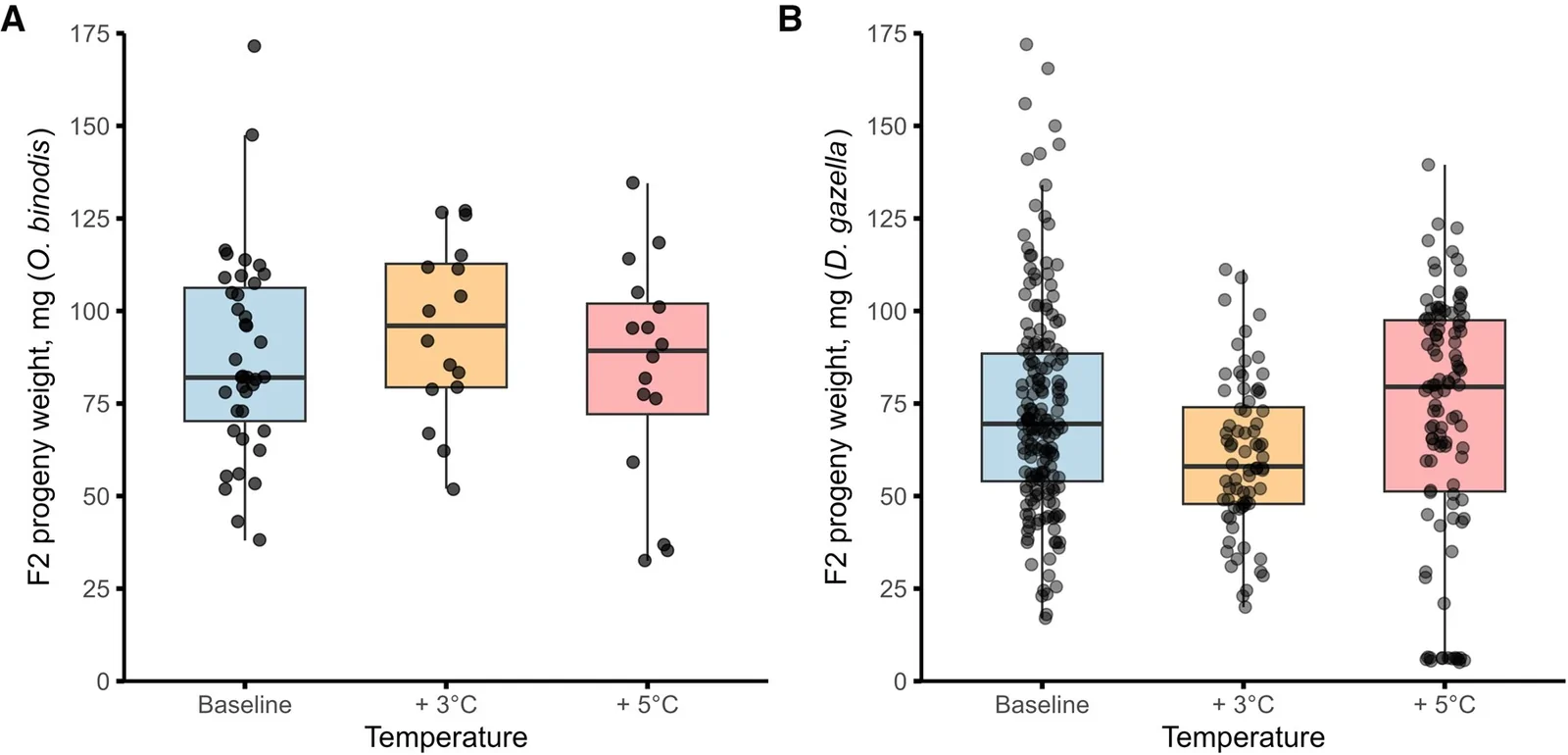
Effect of temperature on F2 progeny body weight (mg) of *Onthophagus binodis* (A) and *Digitonthophagus gazella* (B). Boxplots show the distribution of F2 progeny body weight, and points represent individual F2 beetles. For *O. binodis*, χ^2^ = 1.539, df = 2, and *P *= 0.463; and for *D. gazella*, χ^2^ = 3.929, df = 2, and *P *= 0.140.

### Dung Burial

Dung burial was affected by temperature (Fig. 8), which was 96 g/kg dry matter higher (χ^2^ = 9.769, df = 2, *P *= 0.008) when F1 beetles were reared in the baseline chamber (311 g/kg dry matter) compared to those reared in the +3 °C chamber (215 g/kg dry matter); however, those beetles reared with the highest temperature treatment had an intermediate value, with an average dung burial of 243 g/kg dry matter. Similarly, we found an effect of the feeding week on dung burial across the treatments. For instance, dung burial was 107 g/kg dry matter higher (χ^2^ = 17.119, df = 1, *P *< 0.001) in week 2 than in week 1, although dung burial did not differ (*P *> 0.05) between treatments in week 2. No interaction (χ^2^ = 1.439, df = 2, *P *= 0.487) was observed between temperature and the feeding week. In addition, the relationship between brood number and dung burial was not significant under any temperature treatment during either week 1 or week 2 (Fig. 9A and B; all *P *> 0.20), although the strength and direction of the relationships varied among treatments.

**Fig. 8.**
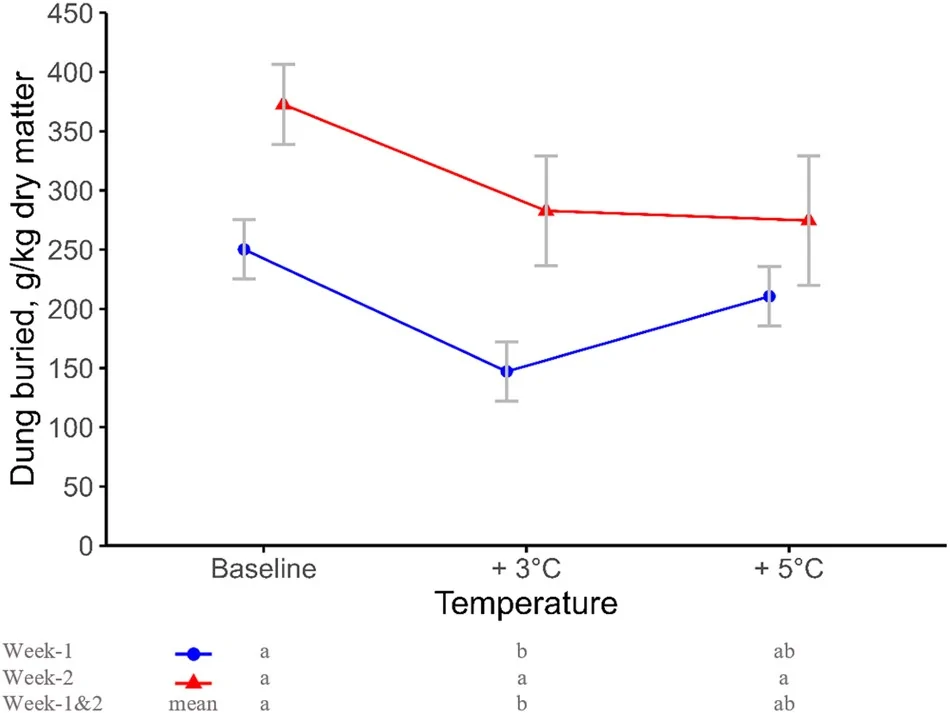
Effects of temperature on dung burial by 1 pair of *Onthophagus binodis* and 2 pairs of *Digitonthophagus gazella*. Error bars indicate the lower and upper bounds of the 95% confidence intervals for dung buried in each feeding week. Different letters below the x-axis title indicate statistically significant differences (*P* < 0.05) for the feeding period. Effect of temperature, χ^2^ = 9.769, df = 2, and *P *= 0.008; effect of feeding week, χ^2^ = 17.119, df = 1, and *P* < 0.001; interaction between temperature and feeding week, χ^2^ = 1.439, df = 2, and *P *= 0.487.

**Fig. 9.**
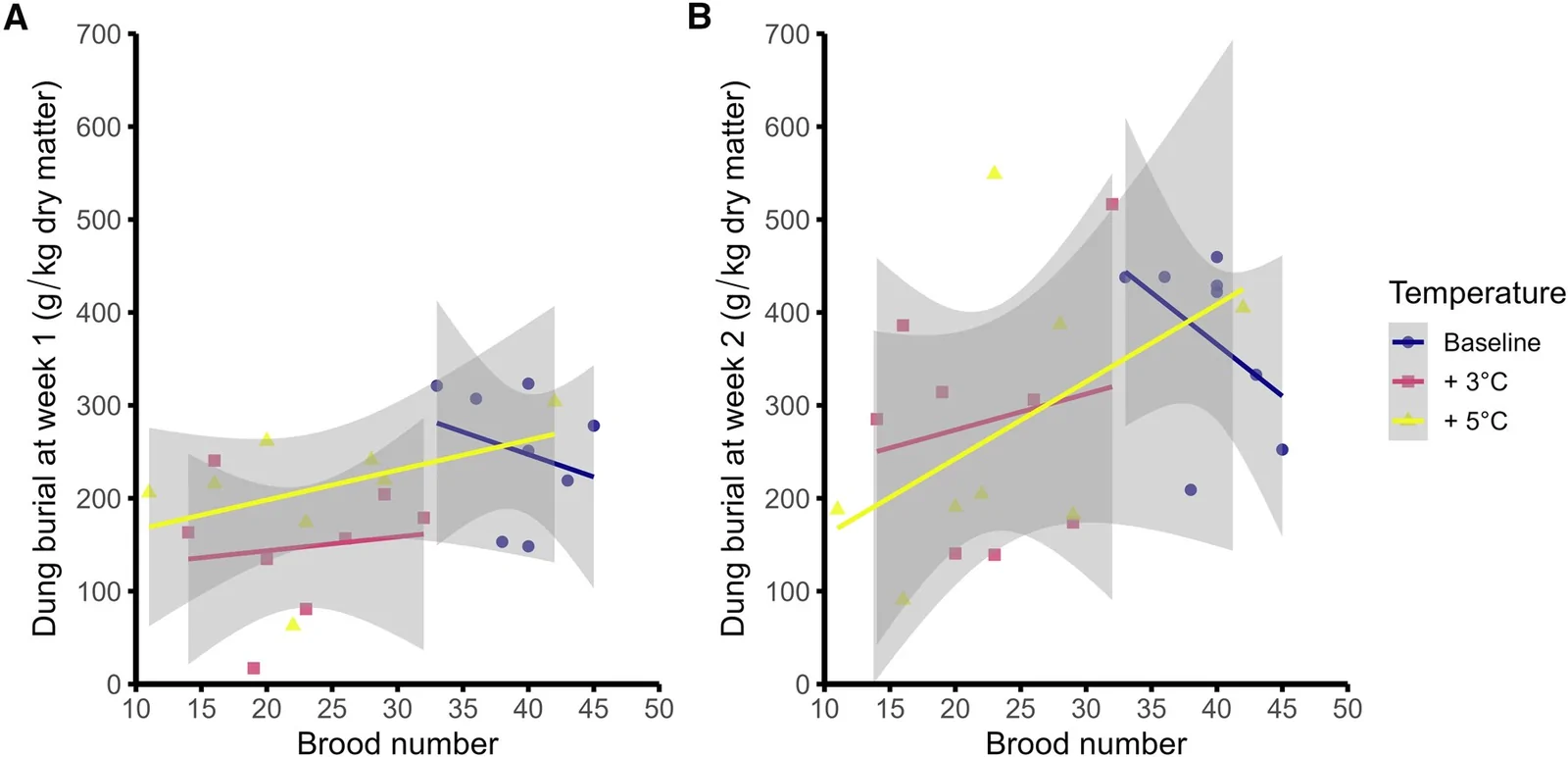
Relationship between the amount of dung buried (g/kg dry matter) and the number of constructed broods by both species of dung beetles (*Onthophagus binodis* and *Digitonthophagus gazella*) under 3 different temperature treatments. (A) Dung burial at the feeding period week 1 and (B) dung burial at the feeding period week 2 in relation to the number of broods. Points represent individual experimental chambers, and colored lines represent treatment-specific linear regressions with 95% confidence intervals (shaded areas). For week 1 (A), the regression equations were y = 440.859 − 4.843x (baseline; R^2^ = 0.067, *P *= 0.537), y = 113.631 + 1.495x (+3 °C; R^2^ = 0.018, *P *= 0.753), and y = 133.577 + 3.226x (+5 °C; R^2^ = 0.181, *P *= 0.293). For week 2 (B), the regression equations were y = 811.387 − 11.142x (baseline; R^2^ = 0.192, *P *= 0.277), y = 196.094 + 3.874x (+3 °C; R^2^ = 0.035, *P *= 0.658), and y = 76.323 + 8.305x (+5 °C; R^2^ = 0.256, *P *= 0.201).

## Discussion

Our study demonstrates that elevated nest temperatures severely impair dung beetle reproduction and functional performance, suggesting that climate warming poses a direct threat to the ecosystem services that these species provide (McConnell et al. 2026). First, elevated temperatures substantially reduce brood production in both species, though offspring survival to adulthood remained unaffected. Second, heat stress significantly suppressed the functional capacity of beetles to bury dung. Third, extreme warmth accelerated progeny development in *D. gazella* but caused divergent morphological responses in adult body size between the 2 species. Together, these findings indicate that while dung beetles may morphologically and developmentally adjust to warmer nest environments, their overall reproductive output and functional efficiency are critically bottlenecked by heat stress.

The present study builds on previous studies in the following ways: (a) we assessed interspecific competition under thermal stress using laboratory-reared mature F1 beetles (*O. binodis* and *D. gazella*) rather than field-collected wild beetles (Holley and Andrew 2019b, 2020, Gotcha et al. 2022); (b) we examined the effects of increased temperature by rearing beetles in temperature-controlled chambers for a period of 2 weeks, compared to previous short-term periods (Holley and Andrew 2019a, Mamantov and Sheldon 2021, Gotcha et al. 2022); (c) we investigated the thermal effects on developmental days, brood success rate, and body physiology of *O. binodis* and *D. gazella* offspring (F2), along with ecosystem services, brood construction, and survivability of adult F1 beetles. Increasing the nest temperature by +3 °C or +5 °C above the average 24-hour baseline temperature (control) induced heat stress in beetles and reduced brood production, dung burial, and the development time of F2 *D. gazella*, indicating that the reared species are susceptible to climate change.

Our work demonstrates that mature F1 beetles of both species can survive in the elevated housing temperatures, suggesting that their body physiology exhibits considerable tolerance to increased chamber temperature. Previous field and laboratory-based trials have shown that dung beetles can adapt to fluctuating temperatures that may vary widely over the course of a day (Holley and Andrew 2019a, 2019b, 2020), which is aligned with our current findings. However, we used mature male and female F1 beetles of known ages (3 pairs) rather than field-collected mass density wild beetles in temperature-controlled chambers, indicating that both wild and laboratory-reared beetles had the capacity to acclimate or tolerate the elevated chamber temperature up to +5 °C. In addition, we housed beetles in a temperature-controlled chamber for 14 days, which is mostly similar to a study by Holley and Andrew (2019a, 2020) who exposed tunnelling or rolling dung beetles to a fluctuating chamber temperature (+2 °C and +4 °C offset to the average 24 hour baseline) for 10 to 12 days. In contrast, Gotcha et al. (2022) housed 3 telecoprid beetle species (*Allogymnopleurus indigaceous*, *Scarabaeus zambezianus*, and *Khepher prodigiosus*) with 3 different densities (1, 2, and 4) and fed 3 dung masses (100 g, 200 g and 400 g) at 3 set temperatures (21 °C, 28 °C and 35 °C) for only a 12 hour photoperiod, which is too short to evaluate the temperature effect on adult survivability. Gotcha et al. (2022) suggested that the survivability of dung beetles is dependent on beetle species, but is also related to their body size, metabolic rate, and physiology. Likewise, Holley and Andrew (2019b) reported that the roller beetles (*Sisyphus rubrus*) had a greater probability of surviving than tunnellers (*Euoniticellus fulvus*) when exposed to a diurnally fluctuating (+2 °C and +4 °C offset) baseline temperature. We used tunnellers and did not observe any impact of thermal stress on the survivability of either species, which may be linked to 2 possible reasons: either adult beetles may remain inactive during the hottest parts of the day by tunnelling into the soil or dung pads, and conserve energy for the rest of the day’s activities (Holley and Andrew 2020); or they may be active under elevated temperature by upregulating heat-shock proteins, which can help beetles to be metabolically functional by protecting against cellular damage (Sgrò et al. 2016). However, we did not observe the diurnal behavior or metabolic function of the surviving beetles, which requires further investigation.

Thermal stress may suppress metabolic and ecological functions, leading not only to reduced dung burial but also to limiting the resources for the next generation of larvae. Our finding aligns with the study conducted by Holley and Andrew (2020) who reported a reduction in dung burial when *O. taurus* was reared in their warmest chamber (+4 °C offset) relative to the control for a 12-day feeding period. However, Holley and Andrew (2020) did not find any effect of warming temperatures on dung burial in another trial, and the difference between the 2 trials in dung burial could be attributed to the age or size of the beetles. Similarly, Gotcha et al. (2022) reported a positive correlation between the body size of telecoprid beetles and the amount of dung buried under thermal stress. In general, large-sized beetles can increase dung burial rates more efficiently than small ones (Nervo et al. 2014). However, such a scenario is not always concrete for F1 beetles, and our previous findings (data under revision) suggest that the rate of dung removal by tunnelling F1 beetles does not depend only on the body size but also on the beetles’ physiology. Therefore, the reduced dung burial might be associated with either drying of the dung under warming temperature/low humidity inside the chamber (Wu et al. 2011) or by decreased activity of beetles to protect their respiratory or cuticular water loss (Terblanche et al. 2010). Previous studies have shown that the desiccation stress of insects is strongly related to high ambient temperature (Addo-Bediako et al. 2001, Gibbs et al. 2003) and beetles with a larger body size can reduce stress due to greater water reserves.

In contrast, Gotcha et al. (2022) reported a significantly higher dung burial rate as the chamber temperature increased for *A. indigaceous* and *S. zambezianus* beetles. The increase in dung utilization with elevated temperature is not surprising because the performance of insects under thermal stress depends on the intensity and duration of exposure to temperature (Oliveira et al. 2021). The study conducted by Gotcha et al. (2022) examined the rate of dung burial by 3 telecoprid species under thermal stress for only a 12-hour period. Such a short duration of temperature may improve heat tolerance through phenotypic plasticity (Sgrò et al. 2016), which allows beetles to acclimate rapidly to the tested temperatures, leading to further continuation of ecosystem services (Gotcha et al. 2021, 2022). The adaptability of dung beetles to thermal stress was also evidenced in our current study, as we observed that the dung burial rate, regardless of thermal treatments, was higher in the second week than in the first 7-day feeding period (Fig. 8). The higher rate of dung burial could be associated with the increased metabolic rate of beetles after 7 days of thermal tolerance (Machekano et al. 2021). In addition, the rate of dung burial during the second week of the feeding period became more inconsistent under thermal conditions (+3 °C and +5 °C) than those reared in the baseline temperature (Fig. 9B; SD = 122.2 and 144.4 vs. 89.8, respectively), also suggesting that the cyclic heat stress induced competition between beetles (deCastro-Arrazola et al. 2023), ultimately leading to individual metabolomic variations on dung utilization, requires detailed investigation for such variations on dung removal.

The reduction in brood construction at elevated chamber temperature indicates that the reproductive behavior of F1 dung beetles is more sensitive to thermal fluctuations. Construction of broods requires energy for continuous digging, rolling and shaping of dung within the chamber (Adam and Piętka 2018, deCastro-Arrazola et al. 2023) which can be compromised under thermal stress, leading to a relocation of energy toward maintaining homeostasis rather than investing in a high-energy-demanding task like foraging and brood production (Kirkpatrick and Sheldon 2022, Fleming et al. 2024). Reduction in brood number under thermal stress was also linked with decreased dung burial, as we observed a significant positive relationship between the amount of dung buried and the number of broods produced (Fig. 9).

In line with our present findings, Holley and Andrew (2020) reported a significant reduction in brood number when tunnelling beetles (*O. taurus*) were housed in the +2 °C or +4 °C chamber compared to those reared in the +0 °C chamber. However, using similar treatments, Holley and Andrew (2019a) reported no effect on the number of broods produced by ball-rolling dung beetles, indicating that the construction of the broods could be species-dependent under such thermal conditions. In contrast, Kirkpatrick and Sheldon (2022) reported an improvement in brood production by female *P. vindex* beetles in a greenhouse treatment, where the mean temperature was increased by +2 °C compared to the baseline temperature. The reason for the increase in brood number at +2 °C offset might be associated with thermal tolerance (Gotcha et al. 2021) or behavioral responses to a warming climate (Mamantov and Sheldon 2021).

The highest-temperature treatment increased the pronotum width of *D. gazella*, which is closely linked with development days. For instance, Rohner and Moczek (2021) reported a greater pronotum width (6.48 ± 0.06 mm) in adult *D. gazella* when the beetle had the shortest juvenile development time (24.7 ± 0.23 days). A similar negative relationship was also reported for *Liatongus militaris*, where the pronotum width was short (5.12 ± 0.02 mm) but had a longer development time (45.6 ± 0.45 days; Rohner and Moczek 2021). In line with the findings of Rohner and Moczek (2021), we observed negative relationships between development days and pronotum width in F2 beetles, although their strength varied among temperature treatments (Fig. 6). The strongest relationship was observed in *D. gazella* under the baseline treatment, while *O. binodis* showed a significant negative relationship only under +5 °C. Alternatively, the highest chamber temperature decreased the emergence time or juvenile development of F2 *D. gazella*, which is in agreement with Esquivel-Román et al. (2025) who demonstrated that increasing the temperature from 29 °C to 33 °C reduced the emergence time of tunnelling *Euoniticellus intermedius* dung beetles. The reason for decreased emergence time could be attributed to the accelerated metabolic and developmental process in *D. gazella* larvae at the highest chamber temperature, which reduces the time required for pupation. In contrast, the highest chamber temperature significantly reduced the pronotum width of *O. binodis* (F2) but did not affect the development days, suggesting that the relationship between development days and pronotum width of F2 beetles under thermal conditions is also species dependent (Rohner and Moczek 2021) and needs further investigations using different densities of F1 beetles under fluctuating ambient temperatures.

The present investigation was limited to a constant density compared to a natural field setting under elevated thermal conditions; however, future work is required to explore how multiple species at varying ages and densities can acclimate to specific or fluctuating temperatures, thereby better understanding interspecific competition over a more extended period of feeding time. In addition, physiological investigations of surviving beetles at the molecular level, such as the expression of heat shock proteins or changes in metabolic rate, could provide valuable insights into the observed behavioral trade-offs.

## Conclusion

Our findings reveal that climate warming poses a direct threat to the reproductive success and functional performance of dung beetles. Because heat stress simultaneously compromises brood construction and dung burial, rising temperatures are poised to bottleneck the vital ecosystem services these species provide. Protecting thermally sensitive habitats is therefore critical to sustaining agricultural nutrient cycling under rapidly changing climatic regimes.

## Supplementary Material

nvag104_Supplementary_Data

## Data Availability

The data and all the statistical analysis models are available online in figshare: https://doi.org/10.6084/m9.figshare.31325461.v3.

## References

[nvag104-B1] Adam B , Piętka J. 2018. Dung beetles and their role in the nature. Edukacja Biologiczna i Środowiskowa. 66:17–26.

[nvag104-B2] Addo-Bediako A , Chown SL, Gaston KJ. 2001. Revisiting water loss in insects: a large scale view. J. Insect Physiol. 47:1377–1388. 10.1016/S0022-1910(01)00128-7 12770144

[nvag104-B3] Andrew NR. 2011. Population dynamics of insects: impacts of a changing climate. In: Rohde K, editor. The balance of nature and human impact. Cambridge University Press. p. 311–324. 10.1017/CBO9781139095075.028

[nvag104-B4] Andrew NR , Hill SJ. 2017. Effect of climate change on insect pest ­management. In: Coll M, Wajnberg E, editors. Environmental pest management: Challenges for Agronomists, Ecologists, Economists and Policymakers, 1st ed. John Wiley and Sons Ltd. pp. 197–215. 10.1002/9781119255574.ch9

[nvag104-B5] AOAC International. 2012. Official methods of analysis. 19th ed. Association of Official Analytical Chemistry.

[nvag104-B6] Bensharada M , Telford R, Stern B, et al 2022. Loss on ignition vs. thermogravimetric analysis: a comparative study to determine organic matter and carbonate content in sediments. J. Paleolimnol. 67:191–197. 10.1007/s10933-021-00209-6

[nvag104-B7] Bhagarathi LK , Maharaj G. 2023. Impact of climate change on insect biology, ecology, population dynamics, and pest management: a critical review. World J. Adv. Res. Rev. 19:541–568. 10.30574/wjarr.2023.19.3.1843

[nvag104-B8] Birkett AJ , Blackburn GA, Menéndez R. 2018. Linking species thermal tolerance to elevational range shifts in upland dung beetles. Ecography. 41:1510–1519. 10.1111/ecog.03458

[nvag104-B9] Bogoni JA , Hernandez MI. 2014. Attractiveness of native mammal’s feces of different trophic guilds to dung beetles (Coleoptera: Scarabaeinae). J. Insect Sci. 14:1–7. 10.1093/jisesa/ieu161 PMC5657881 25528749

[nvag104-B10] Bowler K , Terblanche JS. 2008. Insect thermal tolerance: what is the role of ontogeny, ageing and senescence? Biol. Rev. Camb. Philos. Soc. 83:339–355. 10.1111/j.1469-185X.2008.00046.x 18979595

[nvag104-B11] Brown J , Scholtz CH, Janeau JL, et al 2010. Dung beetles (Coleoptera: Scarabaeidae) can improve soil hydrological properties. Appl. Soil Ecol. 46:9–16. 10.1016/j.apsoil.2010.05.010

[nvag104-B12] Chowdhury ZJ , Schaerf T, Arriaga-Jimenez A, et al 2024. Does inter-species density influence brood ball production in dung beetles? - conferences.com.au. In: The 2024 Australian Entomoligcal Society 55th Annual General Meeting and Scientific Conference. Australian Entomological Society. p. 01. https://conferences.com.au/does-inter-species-density-influence-brood-ball-production-in-dung-beetles/

[nvag104-B13] Chowdhury ZJ , Hemmings Z, Arriaga‐Jimenez A, et al 2026. Combined Effects of Beetle Density and Sex Ratio on Reproductive Trade‐Offs and Dung Burial in Tunnelling Dung Beetles. Ecol. Evol. 16:e74499. 10.1002/ece3.74499 PMC13674177 42890749

[nvag104-B14] Cotter SC , Ward RJS, Kilner RM. 2011. Age-specific reproductive investment in female burying beetles: independent effects of state and risk of death. Funct. Ecol. 25:652–660. 10.1111/j.1365-2435.2010.01819.x

[nvag104-B15] deCastro-Arrazola I , Andrew NR, Berg MP, et al 2023. A trait-based framework for dung beetle functional ecology. J. Anim. Ecol. 92:44–65. 10.1111/1365-2656.13829 PMC10099951 36443916

[nvag104-B16] Dell AI , Pawar S, Savage VM. 2014. Temperature dependence of trophic interactions are driven by asymmetry of species responses and foraging strategy. J. Anim. Ecol. 83:70–84. 10.1111/1365-2656.12081 23692182

[nvag104-B17] Doube BM. 2018. Ecosystem services provided by dung beetles in Australia. Basic Appl. Ecol. 26:35–49. 10.1016/j.baae.2017.09.008

[nvag104-B18] Esquivel-Román A , Baena-Díaz F, Bustos-Segura C, et al 2025. Synergistic effects of elevated temperature with pesticides on reproduction, development and survival of dung beetles. Ecotoxicology. 34:207–218. 10.1007/s10646-024-02825-0 39521745

[nvag104-B19] Fleming JM , Marshall KE, Coverley AJ, et al 2024. Diurnal temperature variation impacts energetics but not reproductive effort across seasons in a temperate dung beetle. Ecology. 105:e4232. 10.1002/ecy.4232 38290131

[nvag104-B20] Genier F , Moretto P. 2017. Digitonthophagus Balthasar, 1959: taxonomy, systematics, and morphological phylogeny of the genus revealing an African species complex (Coleoptera: Scarabaeidae: Scarabaeinae). Zootaxa. 4248:1–110. 10.11646/zootaxa.4248.1.1 28610037

[nvag104-B21] Gibbs AG , Fukuzato F, Matzkin LM. 2003. Evolution of water conservation mechanisms in Drosophila. J. Exp. Biol. 206:1183–1192. 10.1242/jeb.00233 12604578

[nvag104-B22] Giménez Gómez VC , Verdú JR, Casanoves F, et al 2022. Experimental analysis of inter- and intraspecific competition in dung beetle communities. Ecol. Entomol. 47:503–514. 10.1111/een.13135

[nvag104-B23] Gotcha N , Cuthbert RN, Machekano H, et al 2022. Density-dependent ecosystem service delivery under shifting temperatures by dung ­beetles. Sci. Total Environ. 807:150575. 10.1016/j.scitotenv.2021.150575 34634717

[nvag104-B24] Gotcha N , Machekano H, Cuthbert RN, et al 2021. Heat tolerance may determine activity time in coprophagic beetle species (Coleoptera: Scarabaeidae). Insect Sci. 28:1076–1086. 10.1111/1744-7917.12844 32567803

[nvag104-B25] Hanski I , Cambefort Y. 1991. Dung Beetles in Tropical Savannas. In: Hanski I, Cambefort Y, editors. Dung beetle ecology. 1st ed. Princeton University Press. p. 156-178. https://www.jstor.org/stable/j.ctt7zv085.27%0AJSTOR

[nvag104-B9348598] Hartig F , Hartig MF. 2017. Package “DHARMa.” In Vienna, Austria: R Development Core Team. https://cran.r-project.org/web/packages/DHARMa/vignettes/DHARMa.htm

[nvag104-B26] Heiri O , Lotter AF, Lemcke G. 2001. Loss on ignition as a method for estimating organic and carbonate content in sediments: reproducibility and comparability of results. J. Paleolimnol. 25:101–110. 10.1023/A:1008119611481

[nvag104-B27] Hemmings Z , Andrew N, Hall G. 2019. The thermal games dung beetles play

[nvag104-B28] Holley J , Brummell M, Andrew NR. 2026. Introduced dung beetles suppress methane emissions from cattle dung and alter the temporal dynamics of greenhouse gas flux. Ecol. 51:1025–1035. 10.1111/een.70112

[nvag104-B29] Holley JM , Andrew NR. 2019a. Experimental warming disrupts reproduction and dung burial by a ball-rolling dung beetle. Ecol. Entomol. 44:206–216. 10.1111/een.12694

[nvag104-B30] Holley JM , Andrew NR. 2019b. Experimental warming alters the relative survival and emigration of two dung beetle species from an Australian dung pat community. Austral Ecol. 44:800–811. 10.1111/aec.12750

[nvag104-B31] Holley JM , Andrew NR. 2020. Warming effects on dung beetle ecosystem services: brood production and dung burial by a tunnelling dung beetle, Onthophagus taurus (Coleoptera: Scarabaeidae), is reduced by experimental warming. Aust. Entomol. 59:353–367. 10.1111/aen.12448

[nvag104-B32] Hunt J , Simmons LW. 2001. Status-dependent selection in the dimorphic beetle Onthophagus taurus. Proc. R Soc. Lond. B. 268:2409–2414. 10.1098/rspb.2001.1758 PMC1088894 11747558

[nvag104-B33] Kirkpatrick WH , Sheldon KS. 2022. Experimental increases in temperature mean and variance alter reproductive behaviours in the dung beetle Phanaeus vindex. Biol. Lett. 18:20220109–20220122. 10.1098/rsbl.2022.0109 PMC9256084 35857889

[nvag104-B6118800] Lenth R , Singmann H, Love J, Buerkner P, Herve M. 2019. Package ‘emmeans.’ 10.32614/CRAN.PACKAGE.EMMEANS

[nvag104-B875883] R Core Team. 2022. A Language and Environment for Statistical Computing. Vienna: R Foundation for Statistical Computing. https://www.r-project.org/. 10.4236/oalib.1107821

[nvag104-B34] Lima AS , Maciel R, Togni PHB, et al 2024. The functional guilds of dung beetles mediate secondary seed dispersal in a tropical savanna. Appl. Soil Ecol. 204:105736. 10.1016/j.apsoil.2024.105736

[nvag104-B35] Ma CS , Ma G, Pincebourde S. 2021. Survive a warming climate: insect responses to extreme high temperatures. Annu. Rev. Entomol. 66:163–184. 10.1146/annurev-ento-041520-074454 32870704

[nvag104-B36] Machekano H , Zidana C, Gotcha N, et al 2021. Limited thermal plasticity may constrain ecosystem function in a basally heat tolerant tropical telecoprid dung beetle, Allogymnopleurus thalassinus (Klug, 1855). Sci. Rep. 11:22192–22115. 10.1038/s41598-021-01478-x PMC8590042 34772933

[nvag104-B37] Mamantov MA , Sheldon KS. 2021. Behavioural responses to warming differentially impact survival in introduced and native dung beetles. J. Anim. Ecol. 90:273–281. 10.1111/1365-2656.13366 33037612

[nvag104-B38] McConnell NJ , Britton SE, Khanna A, et al 2026. A review on true dung beetles’ evolutionary and ecological responses to temperature and impacts on ecosystem functions. Ecol. Entomol. 51:621–638. 10.1111/een.70088

[nvag104-B39] McConnell NJ , Rohner P. 2025. Environmental modifications of dung beetle larvae shape their growth and life history. J. Exp. Biol. 228:1–10. 10.1242/jeb.251022 PMC12448313 40709487

[nvag104-B40] Menéndez R , Gutiérrez D. 2004. Shifts in habitat associations of dung beetles in northern Spain: climate change implications. Ecoscience. 11:329–337. 10.1080/11956860.2004.11682840

[nvag104-B41] Morgan Fleming J , Carter AW, Sheldon KS. 2021. Dung beetles show metabolic plasticity as pupae and smaller adult body size in response to increased temperature mean and variance. J. Insect Physiol. 131:104215. 10.1016/j.jinsphys.2021.104215 33662376

[nvag104-B42] Nervo B , Tocco C, Caprio E, et al 2014. The effects of body mass on dung removal efficiency in dung beetles. PLoS One. 9:e107699. 10.1371/journal.pone.0107699 PMC4168130 25229237

[nvag104-B43] Nichols E , Spector S, Louzada J, et al 2008. Ecological functions and ecosystem services provided by Scarabaeinae dung beetles. Biol. Conserv. 141:1461–1474. 10.1016/J.BIOCON.2008.04.011

[nvag104-B44] Oliveira BF , Yogo WIG, Hahn DA, et al 2021. Community-wide seasonal shifts in thermal tolerances of mosquitoes. Ecology. 102:e03368. 10.1002/ecy.3368 33866546

[nvag104-B45] Pecl GT , Araújo MB, Bell JD, et al 2017. Biodiversity redistribution under climate change: impacts on ecosystems and human well-being. Science. 355:eaai9214. 10.1126/science.aai9214 28360268

[nvag104-B46] Rohner PT , Moczek AP. 2021. Evolutionary and plastic variation in larval growth and digestion reveal the complex underpinnings of size and age at maturation in dung beetles. Ecol. Evol. 11:15098–15110. 10.1002/ece3.8192 PMC8571579 34765163

[nvag104-B47] Schleuning M , Neuschulz EL, Albrecht J, et al 2020. Trait-based assessments of climate-change impacts on interacting species. Trends Ecol. Evol. 35:319–328. 10.1016/j.tree.2019.12.010 31987640

[nvag104-B48] Sgrò CM , Terblanche JS, Hoffmann AA. 2016. What can plasticity contribute to insect responses to climate change? Annu. Rev. Entomol. 61:433–451. 10.1146/annurev-ento-010715-023859 26667379

[nvag104-B49] Terblanche JS , Clusella-Trullas S, Chown SL. 2010. Phenotypic plasticity of gas exchange pattern and water loss in Scarabaeus spretus (Coleóptera: Scarabaeidae): deconstructing the basis for metabolic rate variation. J. Exp. Biol. 213:2940–2949. 10.1242/jeb.041889 20709922

[nvag104-B50] Urban MC , Tewksbury JJ, Sheldon KS. 2012. On a collision course: competition and dispersal differences create no-analogue communities and cause extinctions during climate change. Proc. R Soc. B. 279:2072–2080. 10.1098/rspb.2011.2367 PMC3311897 22217718

[nvag104-B51] Wu X , Duffy JE, Reich PB, et al 2011. A brown-world cascade in the dung decomposer food web of an alpine meadow: effects of predator interactions and warming. Ecol. Monogr. 81:313–328. 10.1890/10-0808.1

[nvag104-B52] Wu XW , Sun SC. 2012. Artificial warming advances egg-laying and decreases larval size in the dung beetle aphodius erraticus (coleoptera: Scarabaeidae) in a Tibetan alpine meadow. Ann. Zool. Fennici. 49:174–180. 10.5735/086.049.0305

