# Supplementary material for: Thermal stress reduces dung burial, brood production, and the development of dung beetles: a challenge for climate resilience
Source: Environ Entomol. 2026 Nov 5;55(6):nvag104. doi: 10.1093/ee/nvag104 (PMC13739287; doi:10.1093/ee/nvag104)
Supplement: Supplementary file 1 [file nvag104_supplementary_data.docx]

**Thermal stress reduces dung burial, brood production, and the development of dung beetles: A challenge for climate resilience**

**Zinat Jahan Chowdhury, Mohammed Rashed Chowdhury, Zac Hemmings, Karl Vernes, Timothy Martin Schaerf, Nigel R. Andrew, Alfonsina Arriaga Jimenez**

**Highlights**

- Increasing environmental temperatures can disrupt key ecological processes by altering the reproductive performance and functional roles of dung beetles
- Elevated nest temperatures reduced brood production and dung burial, indicating negative effects of heat stress on dung beetle reproductive performance.
- Key life-history traits of F2 progeny, including body weight and brood success, remained largely stable despite increased temperatures.
- Developmental responses varied between species, with faster development in *D. gazella* but no effects in *O. binodis*.
